# Pure red cell aplasia among ABO mismatched hematopoietic stem cell transplant recipients: a 13-years retrospective study and literature review

**DOI:** 10.3389/fonc.2024.1386670

**Published:** 2024-07-02

**Authors:** Elisabetta Metafuni, Maria Teresa Busnego Barreto, Caterina Giovanna Valentini, Sabrina Giammarco, Maria Assunta Limongiello, Federica Sorà, Maria Bianchi, Giuseppina Massini, Nicola Piccirillo, Rossana Putzulu, Filippo Frioni, Andrea Bacigalupo, Luciana Teofili, Patrizia Chiusolo, Simona Sica

**Affiliations:** ^1^ Dipartimento di Diagnostica per Immagini, Radioterapia Oncologica e Ematologia; Fondazione Policlinico Universitario Agostino Gemelli IRCCS, Rome, Italy; ^2^ Hematology and Hemotherapy Service, Hospital Universitario Nuestra Señora de Candelaria, Santa Cruz de Tenerife, Spain; ^3^ Sezione di Ematologia, Dipartimento di Scienze Radiologiche ed Ematologiche, Università Cattolica del Sacro Cuore, Rome, Italy

**Keywords:** PRCA, HSCT, plasma-exchange, isohemagglutinins, rituximab, daratumumab, AB0

## Abstract

**Background:**

Pure red cell aplasia (PRCA) is a possible complication after allogeneic hematopoietic stem cell transplantation (HSCT) with major ABO incompatibility. Patients experience delayed engraftment of the erythroid series, with prolonged transfusion-dependent anemia and iron overload.

**Methods:**

We performed a revision of the most recent literature about post-HSCT PRCA treatment procedures. Moreover, we conducted a retrospective study, over the last 13-years, which included all consecutive major ABO mismatched HSCT performed in our unit, with the aim to assess PRCA incidence, risk factors, and response to different treatments. Overall, 194 patients received a major ABO mismatched transplant from 2010 to 2022. For each patient, data about demographic and transplant characteristics, engraftment, blood transfusion, and possible treatment received were collected.

**Results:**

The literature review returned 23 eligible papers on PRCA treatment, with high success rate using plasma-exchange (PEX) and immunoadsorption procedures, daratumumab, and eltrombopag. Our study identified a total of 24 cases of PRCA. Among risk factors for PRCA development, we have found older recipient age (p=0.01), high pre-HSCT IgG and IgM IHA titer (p<0.0001), major rather than bidirectional ABO incompatibility (p=0.02), low T CD8 lymphocyte count in the graft (p=0.006), relative donor (p=0.02) and bone marrow as stem cell source (p=0.002). However, multivariate analysis confirmed only pre-HSCT IgG IHA titer as the unique risk factor for PRCA occurrence. The optimal cut-off value of pre-HSCT IgG IHA for PRCA development, resulted to be 1/64, with a 100% sensitivity and 68.8% specificity (p<0.0001). All patients with PRCA had received rhEPO and transfusion support and 20 patients received additional treatments like PEX, rituximab, and more recently daratumumab. Comprehensively, PEX and rituximab obtained a response in half of the cases, at a variable time, while the few cases of patients we treated with daratumumab suggest promising results. The overall response rate in our cohort was 75%, with significantly better survival (94.4% vs. 16.7%) and lower transplant-related mortality (6.3% vs. 80%) for PRCA responders.

**Conclusions:**

Standardized guidelines on when and how to treat PRCA are necessary because the current treatment is controversial among centers.

## Scope statement

We have performed a retrospective analysis of the Pure Red Cell aplasia cases we diagnosed in the last 13 years at our Transplant Unit. The scope of the analysis was to identify which risk factors might favor PRCA development among recipients of AB0 mismatched allogeneic stem cell transplantation. The second scope was to evaluate the efficacy of different treatments performed for PRCA management to determine which ones would be the most useful. For this purpose, we also reviewed the literature of the last recent 7 years to highlight the principal treatments mainly used for PRCA management with the relative results in terms of efficacy in PRCA resolution. Currently, a shared algorithm on when and how to treat PRCA patients does not exist, therefore the management of PRCA still remains controversial.

## Introduction

Allogeneic hematopoietic stem cell transplantation (HSCT) is a potentially curative option for many malignant diseases (1). An indispensable element in transplant planning is the selection of a suitable donor in terms of human leukocyte antigen (HLA) match, while ABO compatibility is not mandatory (2). However, the ABO mismatch between recipient and donor must be taken into account as it may lead to acquired pure red cell aplasia (PRCA) (3, 4), with prolonged transfusion requirement and the risk of iron overload. Up to 50% of HSCTs are performed across ABO incompatibility but the influence of this condition on post-transplant outcomes remains controversial (3–5). The incidence of PRCA after AB0 mismatched transplant varies from 10 to 29% of cases (3, 4, 6, 7), and it is observed after major or bi-directional AB0 mismatch. In major AB0 mismatch, accounting for 20–25% of transplants, the recipient showed anti-donor AB0 antibodies, as for a group 0 patient receiving cells from a group A or B donor. In bidirectional AB0 mismatch, which accounts for 5% of transplants, both donor and recipient have antibodies directed against AB0 group antigens of each other, as for group A patient receiving a group B donor or vice versa (4). A picture of PRCA is likely in the presence of persistent anemia and reticulocytopenia for more than 60 days post-HSCT and hypoplasia/aplasia of erythroid precursors in otherwise normal bone marrow (8). The occurrence of PRCA was attributed to the activity of residual recipient plasma cells that continue to produce isohemagglutinins (IHA), albeit in most recipients anti-donor IHA disappeared within 120 days (9, 10).

In this paper, we conducted a retrospective analysis of PRCA cases that occurred after AB0 mismatched HSCT at our Transplant Centre in the last 13 years and we reviewed the recent literature on current treatments of PRCA.

## Methods

### Retrospective study

We conducted a retrospective observational study at the Transplant Unit of Fondazione Policlinico Universitario Agostino Gemelli IRCCS in Rome. All the patients had given written informed consent to use their data for research purposes. The study was conducted according to the Declaration of Helsinki and was approved by the ethics committee of the Fondazione Policlinico Universitario Agostino Gemelli IRCCS in Rome as part of the TOHP (Transplant Outcome in Hematological Patients) study (protocol number 0030921/20).

#### Patients, data collection, definitions, and endpoints

All patients who had received a first stem cell transplant, from January 2010 to December 2022, in the context of major or bidirectional AB0 incompatibility were included. As per institutional guidelines, haematopoietic bone marrow product were treated for red blood cells (RBCs) depletion <1 ml/Kg of the recipient. Among patients with anemia requiring packed red blood cell transfusions at day +60 from HSCT, PRCA was diagnosed when all the following criteria were fulfilled: persistent normochromic and normocytic anemia, reticulocytopenia (absolute count <10*10^9/L and percentage <1%), isolated erythroid aplasia in the bone marrow (11), negative direct antiglobulin test, full leukocyte donor chimerism, normal leukocyte and megakaryocyte hemopoiesis. Anemia cases due to poor graft function, graft failure (12), or early relapsed hematological disease were excluded. Patient’s data were extracted through review of medical records. We collected the following data: recipient and donor age, sex, and AB0 blood group, recipient hematological disease, disease response at transplant, transplant date, hematopoietic cell transplant comorbidity index (HCT-CI) (13), conditioning regimen used, graft-versus-host disease (GvHD) prophylaxis used, donor relationship with recipient, HLA match between donor and recipient, stem cell source, graft composition (CD34, total nucleated cells (TNCs), total mononucleated cells (MNCs), CD3+, CD4+ and CD8+ T lymphocytes count, CD19+ B lymphocytes count), number of packed red blood cell transfusions required from transplant to reticulocyte engraftment, neutrophil, platelet and reticulocyte engraftment, acute GVHD (aGVHD) occurrence and time to aGvHD onset, donor and recipient Cytomegalovirus (CMV) serostatus, CMV DNAemia after transplant, relapse occurrence and time to relapse, death and time to death, cause of death, time to last follow-up. If available, the pre-transplant IHA titers were collected. In patients with PRCA pre- and post-treatment IHA titers were recorded together with treatment performed. PRCA resolution was defined as a stable level of hemoglobin without transfusion requirement and with a stable reticulocyte percentage above 2%. The IHA titers were determined serologically using patient whole blood collected in ethylenediaminetetraacetic acid (EDTA), as previously described (14). Natural IHA (anti-A IgG and IgM, anti-B IgG and IgM) were determined by incubating a 0.8% standard A and B erythrocyte suspension (Diacell AB0 kit, Bio-Rad, California USA) in saline with twofold serial dilutions of plasma, followed by centrifugation. Titers were scored using an anti-human globulin test card (ID-Card Coombs Anti-IgG and ID-Card NaCl, DiaMed, Bio-Rad, California USA). The end point for titration was the highest dilution giving a 1+ reaction. In this paper, we aimed to assess PRCA incidence among patients who had received AB0 mismatched HSCT, with particular interest in risk factors for developing PRCA. Moreover, we looked for possible differences between patients who spontaneously recovered and those who required treatment. Finally, we inquired if different treatments determined different responses.

#### Treatments

One of the main treatments used in our center was plasma-exchange (PEX) either before or after HSCT. Pre-HSCT PEX protocol includes two every other day procedures: replacement of 1 to 1.5 plasma volumes per procedure with 5% albumin was performed to reduce IHA levels before transplantation (15). Post-HSCT PEX protocol includes six every other-day procedures in addition to a double dose (80.000 U) of recombinant human erythropoietin (rhEPO) after the third and the sixth procedures. Other treatments were rhEPO (40.000 IU per week), steroids (1 mg/Kg), antiCD20 monoclonal antibody (rituximab 375 mg/m2/week for 4 weeks), thrombopoietin receptor agonist (eltrombopag 50 to 150 mg per day), and more recently antiCD38 monoclonal antibody (daratumumab 1800 mg subcutaneously weekly for 2 to 8 weeks).

#### Statistical analysis

The cumulative incidence of PRCA was estimated using cumulative incidence analysis, considering death as competing event. The cumulative incidence of aGVHD was estimated using cumulative incidence analysis considering relapse occurrence as competing event. Transplant-related mortality (TRM) was assessed with cumulative incidence analysis, considering relapse occurrence and death for other causes as competing events. Cumulative incidence between groups was compared by applying Fine and Gray’s model. Overall survival (OS) and disease-free survival (DFS) were determined using Kaplan Meier method and log-rank for comparing curves. All patient, graft, and transplant variables were tested for PRCA occurrence using the logistic regression method: recipient and donor age, sex and AB0 incompatibility, IHA titre, underlying hematological disease type and status at transplant, conditioning regimen intensity, GvHD prophylaxis, aGvHD occurrence before day +60 after transplant, graft composition, donor type, HLA match, stem cell source. Only significant variables in univariate analysis were included in multivariate analysis. Patients were divided into two groups according to PRCA status. All patient, graft, and transplant variables were compared between groups using the Mann-Whitney U test for numerical variables and the chi-square tests (Fisher’s exact test was used when the conditions for a chi-square test were not reached) for categorical variables. For IHA titer, Receiving Operator characteristic curves (ROC) were applied to determine the threshold for predicting PRCA occurrence. The statistical analysis was performed with NCSS 10 software. A statistical significance was attributed to a p-value <0.05.

### Literature review

We reviewed published literature on the treatment of PRCA after HSCT with AB0 mismatch in the adult population. The research was performed using PubMed Central (PMC) database, available at the following link https://pubmed.ncbi.nlm.nih.gov/. The keywords used in the research were: PRCA, Pure red cell aplasia, AB0, stem cell transplant, and treatment. The research involved papers published in the last seven years, from 2017 to 2023. Only papers with available full text, case reports or regular articles were considered. Papers on Pediatric patients were excluded.

### Observational study results

We evaluated 194 patients who had received AB0 mismatched HSCT from January 2010 to December 2022. Median follow-up at the observation time fixed in June 2023 was 778 days (95% CI 591–1055). At the observation time, 100 patients (51.5%) were alive after a median of 1723 days (95% CI 1501–2060), and 94 patients (48.5%) died at a median of 406 days after transplant (95% CI 180–318). The cause of death was relapse/progression in 38 cases, secondary neoplasia in one case and transplant-related in 55 cases (intracranial bleeding n=4, myocardial ischemia n=1, heart failure n=2, acute renal failure n=1, pulmonary failure n=3, multi-organ failure n=13, graft failure n=1, GVHD n=5, encephalitis n=3, pneumonia n=5, and sepsis n=17). One- and two-year OS was 68% (95% CI 61.5–74.6) and 57.2% (95% CI 50.1–64.2), respectively. Patients and transplant characteristics are reported in **Table 1**. Recipient/Donor blood groups were as follows: 0/A in 104 couples (53.6%), 0/AB in 7 couples (3.6%), 0/B in 32 couples (16.5%), A/AB in 3 couples (1.6%), A/B in 25 couples (12.9%), B/A in 22 couples (11.3%) and B/AB in one couple (0.5%). At day 30 after transplant neutrophil engraftment (>0.5*10^9/L) was achieved by 88.2% of patients (95% CI 83.7–93) and platelet engraftment was achieved by 71% of patients (95% CI 64.8–77.8). At day 100 after transplant, the cumulative incidence of aGVHD was 39% (95% CI 32.4–46.9) (n=69), graded as follows: 35 grade I (50.7%), 24 grade II (34.8%), 8 grade III (11.6%), and 2 grade IV (2.9%). A total of 59 patients (30.4%) experienced a relapse of the underlying hematological disease with a 1-year and 2-ys DFS of 58.2% (95% CI 51.3–65.2) and 50.6% (95% CI 43.5–57.7), respectively. Finally, 1-year and 2-ys cumulative incidence of TRM was 28.5% (95% CI 22–36.9) and 35.9% (95% CI 28.8–44.7), respectively.

**Table 1:** Characteristics of patients and comparison between groups with or without PRCA.

Variables	All patients (n=194) n (%) or median (95% CI)	PRCA (n=24) n (%) or median (95% CI)	No PRCA (n=170) n (%) or median (95% CI)	P value
Rec age, ys	55 (51–56)	59.5 (55–62)	53 (50–55)	**0.01**
Rec sex F/M	85 (56.2)/109 (43.8)	9 (37.5)/15 (62.5)	76 (44.7)/94 (55.3)	0.5
Diagnosis				
AML/ALL MDS/MPD LPD/PCD	104 (53.6) 62 (32) 28 (14.4)	15 (62.5) 7 (29.2) 2 (8.3	89 (52.3) 55 (32.4) 26 (15.3)	0.6
Status at HSCT				
CR/No CR	88 (45.4)/106 (54.6)	11 (45.8)/13 (54.2)	77 (45.3)/93 (54.7)	1
HLA				
Matched Mismatched Haplo	108 (55.7) 40 (20.6) 46 (23.7)	12 (50) 2 (8.3) 10 (41.7)	96 (56.5) 38 (22.3) 36 (21.2)	0.05
HCT-CI	3 (2–3)	2.5 (2–3)	3 (2–3)	0.8
IHA titre at HSCT				
IgG IgM	1/32 (1/16–1-/32) 1/16 (1/8–1/32)	1/256 (1/128–1/1024) 1/128 (1/32–1/512)	1/16 (1/8–1/32) 1/12 (1/8–1/16)	**<0.0001** **<0.0001**
AB0 incompatibility				
Major Bidirectional	148 (72.3) 46 (23.7)	23 (95.8) 1 (4.2)	125 (73.5) 45 (26.5)	0.02
Graft composition				
CD34*10^9/Kg TNC*10^8/Kg MCN*10^8/Kg CD3*10^6/Kg CD4*10^6/Kg CD8*10^6/Kg CD19*10^6/Kg	5.7 (5.2–6) 6.6 (6.1–7.4) 4.9 (4.7–5.4) 202.6 (165.4–219.1) 125.8 (104.4–132) 52.1 (40.7–65) 44.1 (38–50.1)	5.1 (3.3–6.3) 5.8 (3.2–7.5) 3 (1.6–5.8) 99.5 (28.7–210.9) 58.4 (18–170.5) 18.8 (11.1–38.3) 26.3 (11.8–50.2)	5.8 (5.3–6.1) 6.7 (6.2–7.5) 4.9 (4.8–5.4) 207.4 (170.8–227.2) 128.4 (113.2–141.1) 57 (48.3–67) 44.9 (39–51)	0.2 0.2 0.1 0.05 0.09 **0.006** 0.08
Conditioning				
RIC/MAC	111 (57.2)/83 (42.8)	15 (62.5)/9 (37.5)	96 (56.5)/74 (43.5)	0.6
GvHD prophylaxis				
CsA+MTX CsA+MFA CsA+MFA+PTCy	55 (28.4) 13 (6.7) 126 (64.9)	7 (29.2) 0 (0) 17 (70.8)	48 (28) 13 (8) 109 (64)	0.3
ATG	61 (31.4)	7 (29.2)	54 (31.8)	0.8
Stem cell source				
PB BM CB	141 (72.7) 43 (22.2) 10 (5.1)	12 (50) 12 (50) 0 (0)	129 (75.9) 31 (18.2) 10 (5.9)	**0.002**
Donor				
REL/UD	84 (43.3)/110 (56.7)	16 (66.7)/8 (33.3)	68 (40)/102 (60)	**0.02**
Don sex F/M	64 (33)/130 (67)	7 (29)/17 (71)	57 (34)/113 (66)	0.6
Don age, ys	33 (30–36)	38 (27–47)	33 (3.-36)	0.2
Others				
aGvHD until day +60	61 (31)	7 (29)	54 (32)	0.8
CMV until day +60	62 (32)	8 (33)	54 (32)	0.9
RBC transfusion ¥	8 (6–9)	31.5 (25–40)	6.5 (5–8)	**<0.0001**

Rec, recipient; F, female; M, male; AML, acute myeloid leukemia; ALL, acute lymphoblastic leukemia; MDS, myelodysplatic syndrome; MPD, myeloproliferative disease; LPD, lymphoproliferative disease; PCD, plasma cell disease; CR, complete remission; HSCT, hematopoietic stem cell transplantatio; HCT-CI, hematopoietic cell transplant comorbidity index; IHA, isohemagglutinins titre; CD34, stem cells; TNC, total nucleated cells; MNC, mononucleated cells; CD3, CD3 positive t lymphocytes; CD4, CD4 positive T lymphocytes; CD8, CD8 positive t lymphocytes; CD19, CD19 positive B lymphocytes; RIC, reduced intensity conditioning; MAC, myeloablative conditioning; CsA, cyclosporine A; MTX, methotrexate; MFA, mycofenolic acid; PTCy, post-transplant Cyclophosphamide; ATG, anti-thymocyte globulins; PB, peripheral blood; BM, bone marrow; CB, cord blood; REL, relative; UD, unrelated; ¥, RBC transfusion received until reticulocytes engraftment of death.Bold values are those with statistical significance (p<0.05).

#### PRCA diagnosis and characteristics

At day +60 after HSCT, the cumulative incidence of PRCA was 13.4% (95% CI 9.2–19.5). Twenty-four cases of PRCA were identified in our cohort. In one case the recipient was group B with a group A donor (4.2%). All the other recipients were group 0 with a group A donor in 16 cases (66.6%), a group AB donor in one case (4.2%), and a B group donor in 6 cases (25%). In **Table 1** we reported the comparison of the variables between PRCA group and no PRCA group. Patients of the PRCA group were older compared with no PRCA group (59.5 vs. 53 years, p=0.01). Pre-transplant IHA titers were higher for PRCA group compared with no PRCA group: 1/256 vs. 1/16 for IgG (p<0.0001) and 1/128 vs. 1/12 for IgM (p<0.0001). AB0 incompatibility was mainly major in PRCA group (95.8% vs. 73.5%, p=0.02). Comparing graft composition among groups, patients in the PRCA group had received a low T CD8 lymphocyte amount compared with patients in the no PRCA group (18.8 vs. 57*10^6/Kg, p=0.006). In the PRCA group, the donor was more frequently a relative one (66.7% vs. 40%, p=0.02) and the graft stem cell source was mainly bone marrow (50% vs. 18.2%) or peripheral blood (50% vs. 75.9%) (p=0.002). As expected, the median number of red blood cell transfusions needed until reticulocyte engraftment in the PRCA group was significantly higher compared to no PRCA group (31.5 vs. 6.5, p<0.0001). A trend was identified according to HLA match, with a prevalence of PRCA among HLA haploidentical transplants (41.7% vs. 21.2%) and a minor prevalence among HLA mismatched transplants (8.3% vs. 22.3%) (p=0.05). No differences between the groups were seen when accounting for sex, underlying hematological disease, disease status at transplant, HCT-CI, CD34+, TNC, MNC, T CD3+, T CD8+, and B CD19+ count in the graft, conditioning regimes, GVHD prophylaxis, ATG use, donor age, aGvHD occurrence before day 60, and CMV DNAemia before day 60.

**Figure 1:**
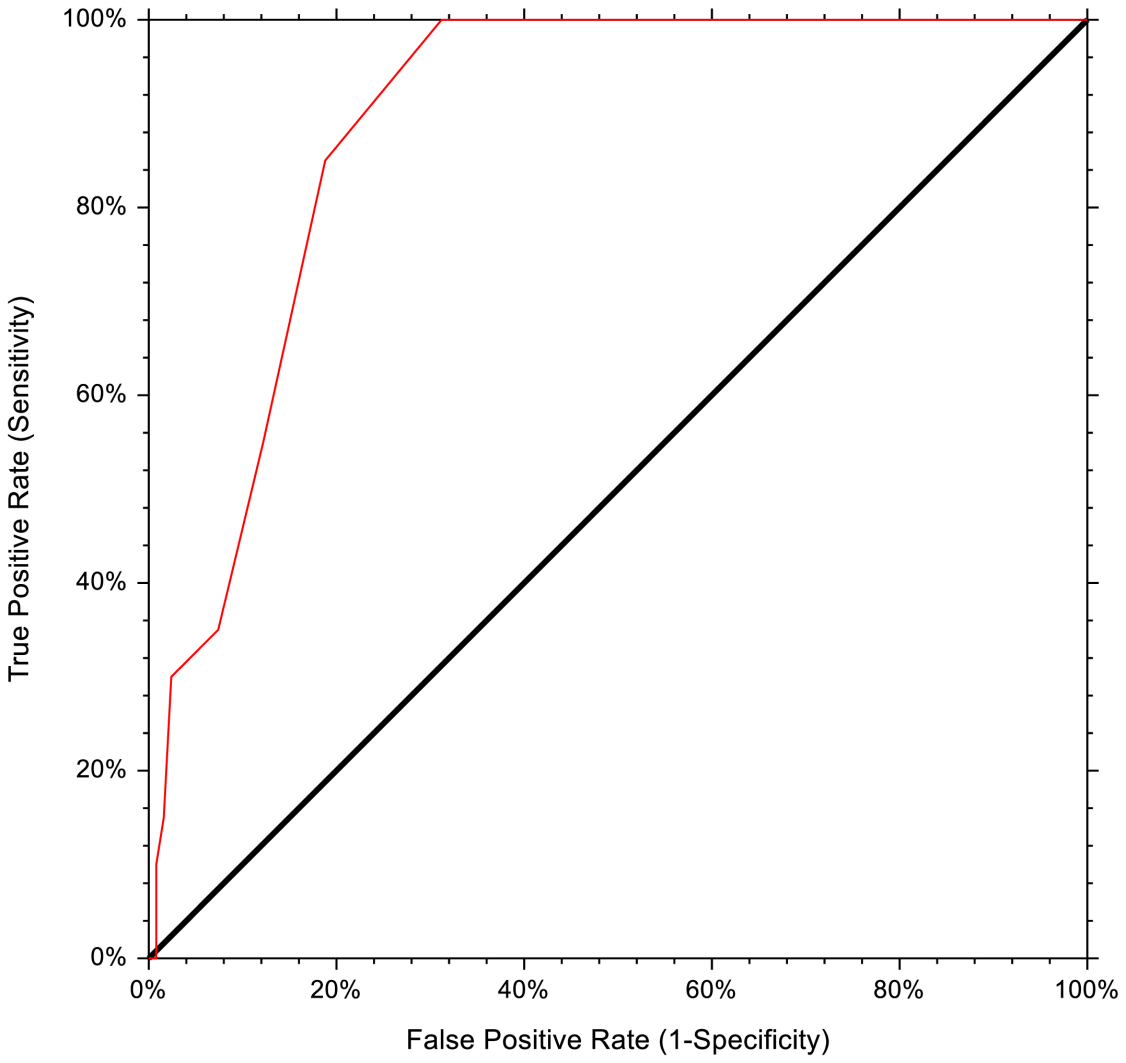
Receiver operating characteristics curve of pre-HSCT IHA titer for PRCA development.

Univariate logistic regression model for PRCA occurrence identify recipient age (OR 1.05, 95% CI 1.01–1.09, p=0.02), major AB0 incompatibility (OR 8.28, 95% CI 1.09–63.10, p=0.04), IgG IHA titer (OR 1.00, 95% CI 1.00–1.00, p=0.04), low CD8+ lymphocytes count in the graft (OR 0.97–0.99, p=0.01), bone marrow stem cell source (OR 4.48, 95% CI 1.84–10.92, p=0.0009) and relative donor (OR 3.0, 95% CI 1.22–7.40, p=0.02) as independent variables. The multivariate logistic regression model confirmed IgG IHA titer as the only significant variable for PRCA occurrence (OR 1.00, 95% CI 1.00–1.00, p=0.02), while a trend returned for recipient age (OR 1.05, 95% CI 0.99–1.11, p=0.05). Using the ROC curve, the pre-transplant IgG IHA cut-off value predictive for PRCA occurrence was 1/64, with 68.8% specificity, 100% sensitivity, 34.5% PPV, and 100% NPV (AUC 0.889, 95% CI 0.821–0.932, p<0.0001; **Figure 1**).

#### PRCA treatment, response, and outcome

**Figure 2:**
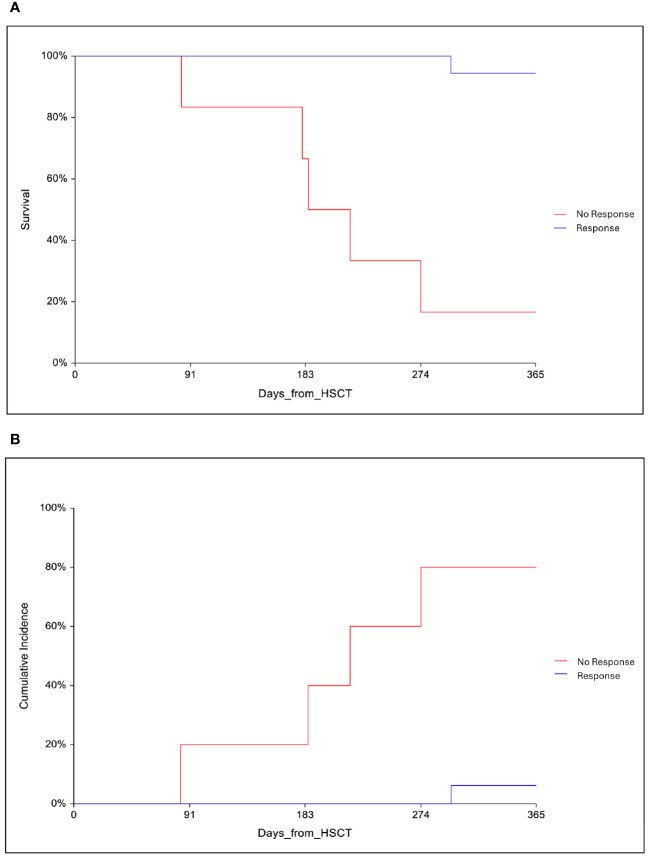
**(A)** One-year overall survival (OS) in responders and non-responders patients with PRCA. **(B)** One-year cumulative incidence of transplant-related mortality in responders and non-responders patients with PRCA.

Ten patients (41.7%) with a high pre-HSCT IHA titer had received 2 pre-HSCT PEX. Nine of them developed PRCA and required further treatment after transplant. All patients received rhEPO started approximately 20 days after HSCT. RhEPO was maintained throughout any specific treatment adopted, until PRCA response. Patients with high level of ferritin had received oral deferasirox 10–20 mg/Kg/day or deferoxamine 20–40 mg/Kg 1–3 times a week, according to initial ferritin level, patients ability to swallow tablets, allergies or renal function. Four patients (16.6%) had not received specific treatment after HSCT for PRCA. Reticulocyte engraftment with transfusion avoidance was achieved at a median of 150 days from transplant (range, 110 to 293) in 3 patients, whereas the last died before obtaining a response. Twenty patients had received a first treatment at a median time of 100 days after transplant (95% CI 75–120): steroids in one case (4.2%), a combination of steroids and rituximab in 7 cases (29.2%), rituximab alone in 6 cases (25%), post-HSCT PEX (6 procedures) with double rhEPO in 5 cases (20.8%), and daratumumab in one case (4.2%). Four patients (20%) had also received eltrombopag (4.2%). Ten patients obtained a response at a median time of 200 days (range, 120 to 282) after HSCT. Two patients died before obtaining a response and the remaining 8 patients needed a second line of treatment started at a median time of 150 days after HSCT (range, 128 to 250): post-HSCT PEX (6 procedures) with double rhEPO in 6 cases (75%), daratumumab in one case (12.5%), and rituximab in one case (12.5%). Three patients obtained a response at a median time of 210 days after HSCT (range, 204 to 308). Two patients died before obtaining a response and the other three patients needed a third line of therapy at a median of 180 days after HSCT (range, 180 to 300): daratumumab in one case, post-HSCT PEX (6 procedures) with double rhEPO in one case, and rituximab in the last case. Two patients obtained a response at 210 and 330 days after HSCT, respectively, whereas the last patient died before obtaining a response. Overall, among 24 patients with PRCA, 8 patients needed two lines of therapy and 3 required three lines of therapy for PRCA. Eighteen patients (75%) obtained a response with reticulocyte engraftment and transfusion avoidance at a median of 207 days after HSCT (95% CI 150–230). Data about PRCA treatment and response are reported in **Table 2**. No predictive variables for response were identified. No treatment-related complications were documented. None of the patients who achieved a response experienced a relapse of PRCA. Comparing outcome variables between PRCA and no PRCA groups, no differences were found in terms of aGVHD occurrence at day 100 (33% vs. 40%, p=0.5), 1-year OS (75 vs. 67%, p=0.9), 1-yr DFS (75% vs. 56%, p=0.4), and 1-year TRM (28 vs. 29%, p=0.8). Considering only the PRCA group, 1-year OS was 94.4% (95% CI 83.9–100) for responders as compared with 16.7% (95% CI 0–46.5) for non-responders (**Figure 2A**). Accordingly, 1-year TRM was 6.3% (95% CI 1–42) for responders and 80% (95% CI 52–100) for non-responders (p<0.0001, **Figure 2B**).

**Table 2:** PRCA cases: treatment and response.

Patient	Donor	Source	Treatment *(starting day, +d)*	Response Y/N
**# 1**	MRD	PB	Pre-HSCT PEX (-3, -1 d), rhEPO (+21 d)	N
**# 2**	MUD	PB	rhEPO (+21 d)	Y
**# 3**	MUD	PB	rhEPO (+21 d)	Y
**# 4**	Haplo	BM	Pre-HSCT PEX (-3, -1 d), rhEPO (+20 d)	Y
**# 5**	MRD	PB	Rh EPO (+20 d), rhEPO + PDN (+30 d)	N
**# 6**	MUD	PB	rhEPO (+21 d), rhEPO + PEX x 6 + PDN (+110 d)	Y
**# 7**	Haplo	BM	Pre-HSCT PEX (-3, -1 d), rhEPO (+20 d), rhEPO + PEX x 6 (+90d)	Y
**# 8**	Haplo	BM	Pre-HSCT PEX (-3, -1 d), rhEPO (+20 d), rhEPO + PEX x 6 + Eltrombopag	Y
**# 9**	Haplo	BM	rhEPO (+20 d), rh EPO + RTX x 4 (+92 d), rhEPO + PEX x 3 (+150 d)	N
**# 10**	MRD	PB	rhEPO (+21 d), rhEPO + RTX x 4 + PDN (+110 d), rhEPO + PEX x 12 (+145 d)	N
**# 11**	MUD	PB	Pre-HSCT PEX (-3, -1 d), rhEPO (+20 d),rhEPO +RTX x 4 (+120 d), rhEPO + PEX x 6 (+150d)	Y
**# 12**	Haplo	BM	Pre-HSCT PEX (-3, -1 d), rhEPO (+21 d), rhEPO + RTX x 4 (+210 d), rhEPO + PEX x 6 + PDN (+250 d)	Y
**# 13**	MMUD	PB	Pre-HSCT PEX (-3, -1 d), rhEPO (+20 d), rhEPO + RTX x 4 (+88 d), rh EPO + PEX x 6 (+128 d), rh EPO + Daratumumab x 8 (+180 d)	Y
**# 14**	Haplo	BM	rhEPO (+20 d), rhEPO + PEX x 6 (+110 d), rhEPO + RTX x 4 (+180 d)	Y
**# 15**	Haplo	BM	rhEPO (+19 d), rhEPO + RTX x 4 (+94 d)	Y
**# 16**	MUD	PB	rhEPO (+19 d), rhEPO + RTX x 4 (+120 d)	Y
**# 17**	Haplo	BM	rhEPO (+20 d), rhEPO + RTX x 4 (+75 d)	Y
**# 18**	Haplo	BM	Pre-HSCT PEX (-3, -1 d), rhEPO (+21 d), rhEPO + RTX x 4 (+100 d)	Y
**# 19**	Haplo	BM	rhEPO (+21 d), rhEPO + RTX x 4 + PDN (+120 d)	Y
**# 20**	MRD	PB	rhEPO (+21 d), rhEPO + RTX x 4 + PDN (+93 d)	N
**# 21**	MRD	PB	rhEPO (+19 d), rhEPO + RTX x 4 + PDN (+110 d)	Y
**# 22**	MUD	BM	rhEPO (+21 d), rhEPO + PEX x 6 (+90 d, +165), rhEPO + RTX x 4 + PDN (+300 d)	Y
**# 23**	MRD	PB	Pre-HSCT PEX (-3, -1 d), rhEPO (+19 d), rhEPO + RTX x 4 (+91 d), rhEPO + Daratumumab x 6 (+130 d)	N
**# 24**	MMUD	BM	Pre-HSCT PEX (-3, -1 d), rhEPO (+21 d), rhEPO + Daratumumab x 2 (+100 d)	Y

MUD, Matched unrelated donor; MMUD, Mismatched unrelated donor; MRD, Matched related donor; Haplo, haploidentical donor; HSCT, Hematopoietic stem-cell transplantation; BM, bone marrow; PB, peripheral blood stem cell; PDN, prednisone; RTX, Rituximab; DLI, rhEPO, erythropoietin; PEX, plasma exchange; d, the day of first administration; m, month of first administration; n, number of patients.

### Results from literature review

**Figure 3:**
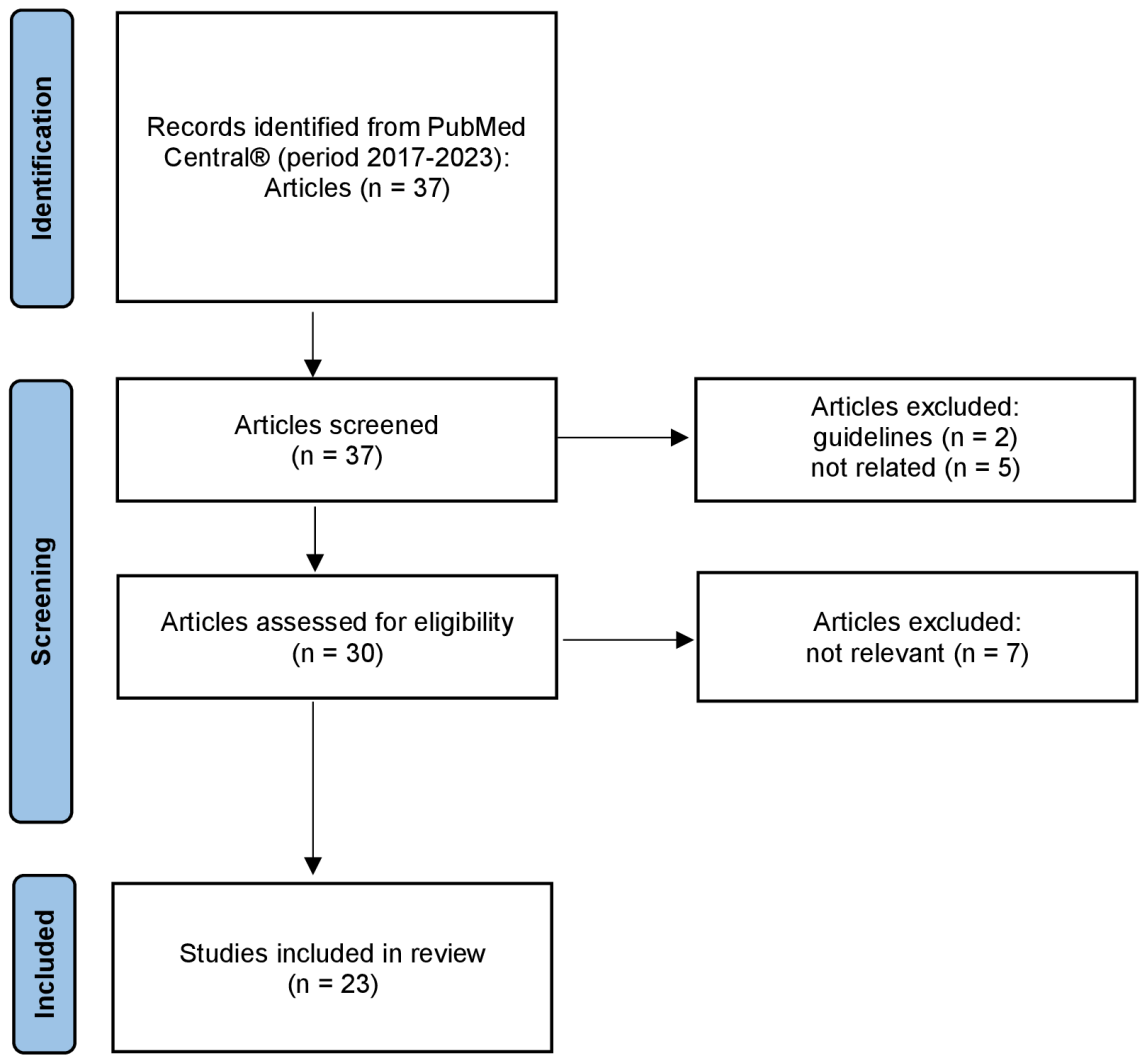
PRISMA flow diagram for the literature review.

Our initial research produced 37 articles (**Figure 3**). Upon first revision of titles and abstracts, 7 articles were excluded: 2 were guidelines and 5 were not related to the treatment of post-HSCT AB0-induced PRCA. During full-text revision, an additional 7 articles were excluded because they were not pertinent within the context of our research. Finally, we analyzed 23 articles. **Table 3** provides a comprehensive summary of therapeutic modalities and their corresponding efficacy parameters across all reviewed cases. A total of 130 cases were identified, all of which exhibited successful resolution, defined as either achieving transfusion independence or an increase in reticulocyte count. Twenty-two patients (17% of cases) required three or more therapeutic lines to achieve a clinical response, while 31% experienced spontaneous remission or recovery without targeted therapeutic intervention. The rest of the patients only required 1 or 2 treatment lines. Some studies included in this review did not provide precise details regarding response times. An analysis of available data revealed variations in the average response times for distinct therapeutic interventions. Three primary modalities for the management of PRCA were used: Daratumumab, Eltrombopag, and apheresis-based therapy like plasma exchange (PEX) and immunoadsorption (IA). These therapeutic interventions were utilized either as monotherapy or as precursors to additional measures. Specifically, from the beginning of the mentioned treatments, patients treated with Daratumumab exhibited an average response time of 26.4 days, while those who had received Eltrombopag therapy demonstrated an average response time of 60 days. PEX and IA exhibited distinct average response times, 28.5 days and 36 days, respectively. Daratumumab, a humanized IgG1-kappa monoclonal antibody targeting CD38, emerges as a focused intervention influencing delayed erythroid engraftment in the context of PRCA. Recent literature underscores the efficacy of Daratumumab in treating nine cases of PRCA (16, 22, 25, 26, 29, 30, 33, 34). Responses were evident following the initial and second doses in three patients (16, 26, 33). Six patients had a prolonged course of PRCA and had undergone extensive previous treatment. Within this cohort, three patients received Daratumumab monotherapy at 205, 270, and 60 days post-HSCT, achieving transfusion independence at 14 days for one patient and 28 days for the remaining two (33, 34). The other patients obtained a response after a median of 33 days (range, 28 to 60) (22, 25, 29, 30). The review included 5 PRCA patients successfully treated with Eltrombopag, an oral thrombopoietin receptor agonist (17, 27, 31). All patients were resistant to multiple lines of treatment, including Rituximab (RTX), Bortezomib, and PEX. In three of these patients, transfusion independence was achieved after 60, 30, and 90 days of treatment (17, 27). No data are available in the remaining patients regarding the exact time of initiation of treatment with Eltrombopag and response. The realm of apheresis therapy presents various procedures, including IA and PEX, designed to eliminate anti-donor IHA. Some authors advocate for IHA reduction through PEX as a preemptive measure before HSCT to prevent the occurrence of PRCA (28, 29). Four patients exclusively subjected to IA using Glycosorb^®^ and an additional two individuals treated with a sequential regimen of IA Glycosorb^®^ followed by PEX and prednisone, on day +159, demonstrated a significant reduction in IHA titer and transfusion independence, achieved within a mean duration of 28.5 days (24). The use of PEX as a therapeutic modality, often in conjunction with other interventions, has been prevalent; however, its efficacy in the context of PRCA treatment varies across reported cases (17–19, 24, 25, 27, 31, 36). In this review, three articles are described in which patients have shown a response following the use of PEX (17, 31, 36). In the first article, two patients developed PRCA refractory to various therapies, leading to the implementation of 10 and 5 series of PEX over 3 and 2 weeks, respectively (36). The second article reports two patients showing a prompt erythroid response after 3 and 5 cycles of PEX, respectively, with one case involving EPO (16). The third article does not specify the number of sessions conducted (31).

**Table 3:** Summary of PRCA in ABO-mismatched allogeneic hematopoietic stem cell transplantation reported in the literature from 2017 to 2023.

Author (year)	*n*	Source	HSCT	Treatment *(n) (+d/+m)*	Response
Chapuy CI (16)	1	PB	MUD	Taper tacrolimus (+105 d), HDG (+209 d), RTX (+235 d), Darbepoetin (+263 d), Daratumumab (+390 d)	TI (+404 d)
Busca A (17)	8	PB	MUD	PEX (1) PEX + EPO (1) EPO (3) PEX+ EPO, RTX (1) EPO (+47 d), PEX (+124 d), RTX (+234 d), Eltrombopag (+365 d) EPO (+47 d), PEX (+112 d), RTX+ EPO (+5 m), Bortezomib (+11 m), Eltrombopag (+16 m)	TI (+425 d, +17 m)
Varela Gómez R (18)	1	BM	MRD	Pre-HSCT IA anti-A (+1 d), Taper CsA, EPO (+1 m), RTX (+10 m), Bortezomib (+18 m), HDG (+22 m)	TI (+24 m)
Tomac G (19)	1	BM	MUD	Pre-HSCT PEX, Prednisone (+470 d) + IVIg (+500 d)	TI (+624 d)
Okamoto K (20)	1	BM	MRD	Conservatively manage, only red blood cell transfusion	TI (+ 2 m)
Nakamura N (21)	1	PB	MRD	Taper CsA (+60 d) → Failure. IL-6 secreted during an infection may have stimulated erythropoiesis (+236 d)	TI (+244 d)
Bathini S (22)	1	PB	MUD	Taper tacrolimus (+68 d) + HDG, RTX (+103 d), Bortezomib (+152 d), Daratumumab (+411 d)	TI (+439 d)
Wada S (23)	5	PB BM	N/A	Resolved spontaneously (3) Withdrawal of tacrolimus (2)	TI (+261 d)
Handisurya A (24)	6	PB	MUD	Darbepoetin alfa/epoetin theta (+55 d) Tapering of IS (+90 d) per-protocol. Antigen-Specific IA with the Glycosorb^®^ only (4) (+159 d) Antigen-Specific IA with the Glycosorb^®^ + PEX + prednisone (2) (+159 d)	IR (+28.5 d after IA)
Salas MQ (25)	1	PB	MRD	RTX (+18 m), HDG (+20 m), PE (+21 m), Bortezomib (+22 m), Daratumumab (+31 m)	TI (+33 m)
Rautenberg C (26)	1	PB	MUD	Taper tacrolimus (+ 63 d), RTX (+77 d), Daratumumab (+206 d)	TI (+216 d)
Gao Y (27)	1	PB	MRD	Taper CsA (+34 d), Prednisone (+34 d), Testosterone (+34 d), EPO (+34 d), PEX (+43 d, +63 d, +119 d and +132 d), DLI (+77 d and +112 d), Eltrombopag (+111 d)	TI (+201 d)
Crysandt M (28)	7	PB	MUD MRD Haplo	Low IHA titre group: Transfusion (1) High-IHA titre group: Transfusion (2) High-IHA titre group: Other methods than selective ABO IA before transplant including PEX (3) High-IHA titre group: Pre-HSCT Selective ABO IA, Rituximab (1)	TI
Henig I (29)	1	PB	MRD	PEX (-3 d), Rituximab (+88 d), Taper until complete discontinuation CsA (+98 d), Bortezomib (+210 d), Daratumumab (+320 d)	TI (+355 d)
Longval T (6)	66	PB BM	MRD/other	Untreated 44 (16 only rhEPO), Specific treatment 22: rhEPO (6) + specific treatment (+70 d) RTX (10) RTX, DLI (5) DLI (2) (+431 d, +236 d) Prednisone (1) (+100 d) Prednisone, RTX (1) (+78 d, +92 d) Romiplostim, RTX (1) (+56 d, +588 d) Boost CD34+ (1) (+326 d) 2nd HSCT (1) (+153 d)	TI
Jeyaraman P (30)	1	PB	MRD	Taper tacrolimus (+60 d), HDG (+74 d), IVIg (+109 d), Bortezomib (+151 d), Daratumumab (+163).	TI (+184 d)
Zhu, P (31)	13	PB	MRD Haplo MUD	Transfusion (4) IVIg + transfusion (3) PEX + transfusion (2) PEX + IVIg + transfusion (2) PEX + RTX + Eltrombopag + Transfusion (1) PEX + DLI + Eltrombopag + Transfusion (1)	TI
Arslan, S (32)	5	PB	MRD MUD	IS withdrawal + prednisone + RTX + ibrutinib (1) IS withdrawal + prednisone + RTX + bortezomib (1) IS withdrawal + prednisone + DLI + RTX + bortezomib + danazole + ibrutinib (1) IS withdrawal + dexamethasone + IVIg + RTX + ibrutinib (1) IS withdrawal + RTX + darbepoetin + ibrutinib (1)	TI
Martino R (33)	2	N/A	N/A	Daratumumab (+205 d, +270 d)	IR (+219 d, 298 d)
Dovern E (34)	1	PB	MRD	Daratumumab (+60 d)	TI (+88 d)
Tavakoli F (35)	1	N/A	MRD	Taper CsA (+50 d), rhEPO (+53 d)	TI (+170 d)
Vivero A (36)	2	PB BM	MRD	LDI (+6 m), RTX (+10 m, +4 m), Daratumumab (+11 m, +5 m), PEX (+12 m, +5,6 m)	TI (+14 m, +6 m)
Jiménez-Ochoa, M (37)	3	N/A	N/A	RTX	RCE (+120 d, +248 d, N/A)

MUD, Matched unrelated donor; MRD, Matched related donor; Haplo, haploidentical donor; HSCT, Hematopoietic stem-cell transplantation; BM, bone marrow; PB, peripheral blood stem cell; HDG, High dose glucocorticoids; IVIg, intravenous immunoglobulins; CsA cyclosporine A; RTX, Rituximab; DLI, Donor lymphocyte infusion; IS, immunosuppression; IHA, isohemagglutinins; rhEPO, erythropoietin; PEX, plasma exchange; IA, immunoadsorption; IL-6, Interleukin 6; TI, Transfusion independence, IR, Increase of reticulocyte; RCE, Red cell engraftment; N/A, not available; d, the day of first administration; m, month of first administration; n, number of patients.

## Discussion

In this paper, we aimed to study PRCA cases that occurred in our transplant unit in the last thirteen years. The purpose of this retrospective analysis was to identify possible risk factors for PRCA occurrence among patients transplanted across AB0 mismatch and to analyze different treatments applied to PRCA cases with relative responses. The incidence of PRCA at day 60 in our cohort was 13.4%, in line with other reports (3, 4, 6, 7). The sole variable resulting from multivariate analysis as predictive for PRCA developed was pre-HSCT IgG IHA, with an optical cut-off value of 1/64 (100% sensitivity and 68.8% specificity). The second point that emerged from our analysis was that pre-HSCT PEX was not found to be an effective procedure to prevent PRCA since it failed in 9 out of 10 cases. The main treatments used at our center for PRCA treatment were post-HSCT PEX, rituximab, and, more recently, daratumumab. Comprehensively, post-HSCT PEX and rituximab had guaranteed a response in half of the cases, at a variable time, while the few cases in which we used daratumumab suggested promising results. The overall response rate in our cohort was 75%, with significantly better survival (94.4% vs. 16.7%) and lower TRM (6.3% vs. 80%) for responders. However, the higher mortality in non-responders may be affected by those patients who died from HSCT-related complications before responding who were assigned to the non-responders group. Nevertheless, it cannot be denied that prolonged immunosuppressive therapy with rituximab, steroids, or daratumumab may increase transplant-related mortality due to a higher risk of infectious complications.

Although the exact pathogenesis of PRCA after HSCT is not fully understood, the hypothesis is that the persistence of host B lymphocytes and plasma cells producing anti-donor IHA is responsible for delayed erythroid engraftment (9). PRCA is more frequently reported after a transplant from a group A donor to a group 0 recipient (4, 38). Other factors reported to increase PRCA risk are reduced intensity conditioning (14), sibling donor (39), and high anti-A IHA titer (40, 41). Regarding the conditioning regimen, it was reported that PRCA occurred more frequently after fludarabine-busulfan conditioning regardless of intensity (42) and less frequently after myeloablative conditioning including cyclophosphamide and total body irradiation (14). In our cohort, no differences in terms of PRCA incidence were observed according to the conditioning regimen. According to donor type, it was reported that a matched related donor is associated with a high risk for PRCA and prolonged persistence of anti-donor IHA when compared to an unrelated donor or a haploidentical one (5, 31, 43–45). In our study, a relative donor, regardless of HLA match, was associated with high PRCA incidence as was for bone marrow source. According to stem cell source, no difference in terms of PRCA was reported between peripheral blood and bone marrow, albeit delayed engraftment was seen after bone marrow HSCT from AB0 mismatched donor (46). Bone marrow hematopoietic cell products contain a high amount of RBCs (25–35% of the total volume) which can cause acute hemolysis during infusion as well as subsequent PRCA. RBC depletion of bone marrow graft is usually performed to reduce donor RBCs infusion to 10–40 ml (15). On the other hand, no cases of PRCA were usually described after cord blood transplant as was in our cohort (6, 23, 47), albeit the low number of cord blood grafts in our cohort (n=10). Although the exact reason for this is still unknown it has been thought that the low expression of A and B antigens on the red blood cells of infants determines a minor hemolysis induced by recipient IHA against donor blood group (23, 48, 49). Moving to the pre-HSCT IHA titer, a recent paper by Lemaire and colleagues (50) analyzed the trend of the IHA titer in a group of patients submitted to HSCT with AB0 incompatibility. Anti-A IHA disappeared spontaneously after a median of 28 days in 82% of the cases. Anti-B IHA spontaneously disappeared after a median of 16 days in 96% of cases. This faster disappearance of anti-B IHA compared with anti-A IHA is also reported elsewhere (40, 51). The prolonged persistence of anti-A IHA together with high anti-A titer might justify the higher incidence of PRCA reported for group 0 recipients of a group A donor (40, 45).

A high titer of IHA before HSCT is associated to an increased risk for PRCA development. In our cohort, the threshold of 1/64 has the best combination of sensitivity and specificity, as previously described by the group of Longval (6). This observation explains why some centers use pre-HSCT PEX or IA to reduce the risk of PRCA, however, results are controversial, and an agreement on the IHA title to be considered high is lacking. Stussi and colleagues reported pre-HSCT PEX alone or combined with red blood cell transfusion according to the donor group as a reliable procedure to reduce the occurrence of PRCA (44). Curley and collaborators reported the results of pre-HSCT PEX combined with donor group secretor plasma infusion after PEX and before stem cell infusion on day 0. In this case, residual IHA titer would be significantly reduced via antigen-antibody binding, and the use of secretor plasma would eliminate the risk of hemolysis that might occur using donor group red blood cell infusion (7). On the contrary, Damodar and colleagues did not find any difference in PRCA occurrence according to pre-HSCT IHA titer, as well as for patients who received PEX with donor plasma group for reducing pre-HSCT IHA titer in bone marrow recipients (52). Also in our cohort, pre-HSCT PEX did not prevent PRCA in patients with high pre-HSCT IHA titer. Finally, opposite to other authors (6, 39), we had not reported an association between early aGVHD occurrence and PRCA development. Mielcarek reported a close relationship between aGVHD occurrence and IHA disappearance after matched related donor HSCT. He postulated that anti-recipient donor T cells would eliminate antibody-producing recipient cells leading to IgG and IgM IHA disappearance. However, they cannot exclude that immunosuppressive therapy against GVHD rather than GVHD itself might contribute to rapid IHA titer abatement (39). To date, a clear correlation between AB0 incompatibility and GVHD is not stated, as is for a correlation between AB0 incompatibility and DFS or TRM (31).

The revision of the literature cases of PRCA who had received a specific therapy reported varying results for the different treatments administered. A comment is due about time to response that favors daratumumab, followed by PEX and IA, and finally eltrombopag.

The main point that needs to be assessed is which patients with PRCA need to be treated, given the possibility of spontaneous recovery within a few months. It is undeniable that patients with prolonged and heavy transfusion needs, at risk of iron overload, and unresponsive to rhEPO could benefit from a specific treatment. EPO-agonist, although widely used for transfusion-dependent anemia, appeared to be not enough to achieve red cell engraftment in patients with erythroid precursor aplasia (6, 17). The role of PEX or IA alone can be useful to rapidly reduce the IHA titer, although the effect might be transient and unable to reverse red cell aplasia (17, 24, 25, 31, 32). The reduction of immunosuppression can be considered as a possible option, enhancing donor T cell activity against donor residual plasma cells, but it should be carefully weighted given the risk of GVHD flare and the variability of responses (16, 21–23, 26, 27, 30, 32). Another option is represented by monoclonal antibodies rituximab and daratumumab. Rituximab is used to remove residual recipient B lymphocytes and the results for rituximab alone or combined with other therapy were frequently satisfying (6, 17, 32, 37). The last entry is daratumumab, targeting host plasma cells sustaining delayed erythroid engraftment guarantees a response even in patients who have previously received more lines of treatment (16, 18, 22, 25, 26, 29, 30, 33, 34, 36).

In conclusion, among patients who developed PRCA, treatment should be considered for those patients who do not spontaneously recover after 4 months. During the watch and wait phase, iron depletion methods together with rhEPO should be applied to avoid iron overload and to stimulate erythroid precursors. When PRCA is prolonged the choice of the most appropriate treatment should be made balancing carefully risks and benefits, with particular attention to infectious and GVHD risks.

## Data availability statement

The data analyzed in this study is subject to the following licenses/restrictions: since the database contains sensitive patient data, the same could be shared under email request to the corresponding author and after anonymization of sensitive data. Requests to access these datasets should be directed to elisabetta.metafuni@policlinicogemelli.it.

## Ethics statement

The studies involving humans were approved by ethics committee of the Fondazione Policlinico Universitario Agostino Gemelli IRCCS, Largo Agostino Gemelli, 8, 00168, Rome, Italy. The studies were conducted in accordance with the local legislation and institutional requirements. The participants provided their written informed consent to participate in this study.

## Author contributions

EM: Conceptualization, Data curation, Formal analysis, Writing – original draft, Writing – review & editing. MBu: Conceptualization, Writing – original draft, Writing – review & editing. CV: Data curation, Writing – review & editing. SG: Data curation, Writing – review & editing. ML: Writing – review & editing. FS: Writing – review & editing. MBi: Writing – review & editing. GM: Writing – review & editing. NP: Writing – review & editing. RP: Writing – review & editing. FF: Data curation, Writing – review & editing. AB: Writing – review & editing. LT: Writing – review & editing. PC: Writing – review & editing. SS: Writing – review & editing.

## References

[1] Passweg JR Baldomero H Bregni M Cesaro S Dreger P Duarte RF. Hematopoietic SCT in Europe: data and trends in 2011. Bone Marrow Transplant. (2013) 48:1161–7. doi: 10.1038/bmt.2013.51 PMC3763517 23584439

[2] Rydberg L. ABO-incompatibility in solid organ transplantation. Transfus Med. (2001) 11:325–42. doi: 10.1046/j.1365-3148.2001.00313.x 11532188

[3] Vo AK Hervig T Reikvam H. Pure red cell aplasia after hematopoietic stem cell transplantation - experimental therapeutic approaches. Expert Opin Investig Drugs. (2022) 31:881–4. doi: 10.1080/13543784.2022.2113055 35975626

[4] Worel N. ABO-mismatched allogeneic hematopoietic stem cell transplantation. Transfus Med Hemother. (2016) 43:3–12. doi: 10.1159/000441507 27022317 PMC4797460

[5] Marco-Ayala J Gómez-Seguí I Sanz G Solves P. Pure red cell aplasia after major or bidirectional ABO incompatible hematopoietic stem cell transplantation: to treat or not to treat, that is the question. Bone Marrow Transplant. (2021) 56:769–78. doi: 10.1038/s41409-020-01124-6 33188257

[6] Longval T Galimard JE Lepretre AC Suarez F Amiranoff D Cazaux M. Treatment for pure red cell aplasia after major ABO incompatible allogeneic stem cell transplantation: a multicentric Study. Br J Haematol. (2021) 193:814–26. doi: 10.1111/bjh.17463 33844842

[7] Curley C Pillai E Mudie K Western R Hutchins C Durrant S. Outcomes after major or bidirectional ABO-mismatched allogeneic hematopoietic progenitor cell transplantation after pretransplant isoagglutinin reduction with donor-type secretor plasma with or without plasma exchange. Transfusion. (2012) 52:291–7. doi: 10.1111/j.1537-2995.2011.03295.x 21848968

[8] Hirokawa M Fukuda T Ohashi K Hidaka M Ichinohe T Iwato K. PRCA Collaborative Study Group. Efficacy and long-term outcome of treatment for pure red cell aplasia after allogeneic stem cell transplantation from major ABO-incompatible donors. Biol Blood Marrow Transplant. (2013) 19:1026–32. doi: 10.1016/j.bbmt.2013.04.004 23583828

[9] Griffith LM McCoy JPJ Bolan CD Stroncek DF Pickett AC Linton GF. Persistence of recipient plasma cells and anti-donor isohaemagglutinins in patients with delayed donor erythropoiesis after major ABO incompatible non-myeloablative haematopoietic cell transplantation. Br J Haematol. (2005) 128:668–75. doi: 10.1111/j.1365-2141.2005.05364.x 15725089

[10] Migdady Y Pang Y Kalsi SS Childs R Arai S. Post-hematopoietic stem cell transplantation immune-mediated anemia: a literature review and novel therapeutics. Blood Adv. (2022) 6:2707–21. doi: 10.1182/bloodadvances.2021006279 PMC9043947 34972204

[11] Means RTJ. Pure red cell aplasia. Blood. (2016) 128:2504–9. doi: 10.1182/blood-2016-05-717140 27881371

[12] Prabahran A Koldej R Chee L Ritchie D. Clinical features, pathophysiology, and therapy of poor graft function post–allogeneic stem cell transplantation. Blood Adv. (2022) 6:1947–59. doi: 10.1182/bloodadvances.2021004537 PMC8941468 34492685

[13] Sorror ML Maris MB Storb R Baron F Sandmaier BM Maloney DG. Hematopoietic cell transplantation (HCT)-specific comorbidity index: a new tool for risk assessment before allogeneic HCT. Blood. (2005) 106:2912–9. doi: 10.1182/blood-2005-05-2004 PMC1895304 15994282

[14] Bolan CD Leitman SF Griffith LM Wesley RA Procter JL Stroncek DF. Delayed donor red cell chimerism and pure red cell aplasia following major ABO-incompatible nonmyeloablative hematopoietic stem cell transplantation. Blood. (2001) 98:1687–94. doi: 10.1182/blood.v98.6.1687 11535498

[15] Connelly-Smith L Alquist CR Aqui NA Hofmann JC Klingel R Onwuemene OA. Guidelines on the use of therapeutic apheresis in clinical practice - evidence-based approach from the writing committee of the american society for apheresis: the ninth special issue. J Clin Apher. (2023) 38:77–278. doi: 10.1002/jca.22043 37017433

[16] Chapuy CI Kaufman RM Alyea EP Connors JM. Daratumumab for delayed red-cell engraftment after allogeneic transplantation. N Engl J Med. (2018) 379:1846–50. doi: 10.1056/NEJMoa1807438 30403942

[17] Busca A Dellacasa C Giaccone L Manetta S Biale L Godio L. Eltrombopag for the treatment of refractory pure RBC aplasia after major ABO incompatible hematopoietic stem cell transplantation. Biol Blood Marrow Transplant. (2018) 24:1765–70. doi: 10.1016/j.bbmt.2018.04.022 29684566

[18] Varela Gómez R Vázquez Vázquez G Noriega Concepción V Galego García A Andón Saavedra C. Successful treatment of pure red cell aplasia with high-dose dexamethasone after ABO-incompatible allogeneic hematopoietic stem cell transplantation. Hematol Oncol Stem Cell Ther. (2018) 11:44–6. doi: 10.1016/j.hemonc.2017.08.004 29079126

[19] Tomac G Bojanić I Mazić S Vidović I Raos M Ćepulić BG. Haemolysis, pure red cell aplasia and red cell antibody formation associated with major and bidirectional ABO incompatible haematopoietic stem cell transplantation. Blood transfus. (2018) 16:397–404. doi: 10.2450/2017.0322-16 28488966 PMC6034778

[20] Okamoto K Osone S Saito T Imamura T Hosoi H. Pure red cell aplasia following the rapid reduction and discontinuation of cyclosporine for mixed chimerism after allogeneic bone marrow transplantation. Rinsho ketsueki. (2018) 59:2408–12. doi: 10.11406/rinketsu.59.2408 30531134

[21] Nakamura N Ninomiya S Matsumoto T Nakamura H Kitagawa J Hara T. Recovery of pure red cell aplasia following hematopoietic stem cell transplantation associated with interleukin (IL)-6 elevation caused by odontogenic infection. Intern Med. (2018) 57:3175–7. doi: 10.2169/internalmedicine.0869-18 PMC6262699 29877260

[22] Bathini S Holtzman NG Koka R Singh Z Wilding E Zou Y. Refractory postallogeneic stem cell transplant pure red cell aplasia in remission after treatment with daratumumab. Am J Hematol. (2019) 94:E216–9. doi: 10.1002/ajh.25515 31120638

[23] Wada S Asano-Mori Y Yamamoto H Yuasa M Kageyama K Kaji D. No post-transplant pure red cell aplasia development in 106 major ABO incompatible cord blood transplantation. Bone Marrow Transplant. (2019) 54:765–8. doi: 10.1038/s41409-018-0375-2 30401968

[24] Handisurya A Worel N Rabitsch W Bojic M Pajenda S Reindl-Schwaighofer R. Antigen-specific immunoadsorption with the glycosorb® ABO immunoadsorption system as a novel treatment modality in pure red cell aplasia following major and bidirectional ABO-incompatible allogeneic hematopoietic stem cell transplantation. Front Med (Lausanne). (2020) 7:585628. doi: 10.3389/fmed.2020.585628 33195341 PMC7642244

[25] Salas MQ Alahmari A Lipton JH. Successful treatment of refractory red cell aplasia after allogeneic hematopoietic cell transplantation with daratumumab. Eur J Haematol. (2020) 104:145–7. doi: 10.1111/ejh.13343 31693245

[26] Rautenberg C Kaivers J Germing U Haas R Ackerstaff S Hoffmann T. Daratumumab for treatment of pure red cell aplasia after allogeneic stem cell transplantation. Bone Marrow Transplant. (2020) 55:1191–3. doi: 10.1038/s41409-019-0664-44 31481799

[27] Gao Y Gao F Shi J Fu H Huang H Zhao Y. Successful treatment of refractory pure red cell aplasia with eltrombopag after ABO-incompatible allogeneic hematopoietic stem cell transplantation. J Zhejiang Univ Sci B. (2021) 22:695–700. doi: 10.1631/jzus.B2000532 34414703 PMC8377576

[28] Crysandt M Soysal H Jennes E Holtick U Mrotzek M Rehnelt S. Selective ABO immunoadsorption in hematopoietic stem cell transplantation with major ABO incompatibility. Eur J Haematol. (2021) 107:324–32. doi: 10.1111/ejh.13668 34022082

[29] Henig I Yehudai-Ofir D Zohar Y Zuckerman T. Pure red cell aplasia following ABO-mismatched allogeneic hematopoietic stem cell transplantation: resolution with daratumumab treatment. Acta Haematol. (2021) 144:683–7. doi: 10.1159/000515257 33887733

[30] Jeyaraman P Borah P Rajput P Dayal N Pathak S Naithani R. Daratumumab for pure red cell aplasia post ABO incompatible allogeneic hematopoietic stem cell transplant for aplastic anemia. Blood Cells Mol Dis. (2021) 88:102464. doi: 10.1016/j.bcmd.2020.102464 32653327

[31] Zhu P Wu Y Cui D Shi J Yu J Zhao Y. Prevalence of pure red cell aplasia following major ABO-incompatible hematopoietic stem cell transplantation. Front Immunol. (2022) 13:829670. doi: 10.3389/fimmu.2022.829670 35222414 PMC8873189

[32] Arslan S Ali H Mei M Marcucci G Forman S Nakamura R. Successful treatment of refractory pure red cell aplasia in major ABO-mismatched allogeneic hematopoietic stem cell transplant with single agent Ibrutinib. Bone Marrow Transplant. (2022) 57:830–3. doi: 10.1038/s41409-022-01590-0 35194155

[33] Martino R García-Cadenas I Esquirol A. Daratumumab may be the most effective treatment for post-engraftment pure red cell aplasia due to persistent anti-donor isohemagglutinins after major ABO-mismatched allogeneic transplantation. Bone Marrow Transplant. (2022) 57:282–5. doi: 10.1038/s41409-021-01507-3 PMC8552208 34711914

[34] Dovern E Biemond BJ Nur E. Case report: Successful treatment with daratumumab for pure red cell aplasia in a patient with mixed lymphoid chimerism after ABO-mismatched stem cell transplant for sickle cell disease. Front Immunol. (2023) 14:1212007. doi: 10.3389/fimmu.2023.1212007 37426651 PMC10326381

[35] Tavakoli F Mohammadian M Ghorbi MD Shahsavan S Halvachi D Parkhideh S. Successful treatment of a pure red cell aplasia patient following ABO-mismatched hematopoietic stem cell transplantation from a sibling donor with multiple sclerosis. Transplant Immunol. (2023) 79:101863. doi: 10.1016/j.trim.2023.101863 37236515

[36] Vivero A Peedin AR Gao Y Karp JK. Successful treatment of pure red cell aplasia using therapeutic plasma exchange after ABO-incompatible hematopoietic stem cell transplant. J Clin Apher. (2023) 38:495–9. doi: 10.1002/jca.22041 36703597

[37] Jiménez-Ochoa MA Contreras-Serratos MM González-Bautista ML López-Macías C Torres-Fierro A Urbina-Escalante E. ABO incompatibility and complications in hematopoietic stem cell transplantation. Rev Med Inst Mex Seguro Soc. (2023) Suppl 1):S12–8. PMC10396064 36378017

[38] Zhu K-e Li J-p Zhang T Zhong J Chen J. Clinical features and risk factors of pure red cell aplasia following major ABO-incompatible allogeneic hematopoietic stem cell transplantation. Hematology. (2007) 12:2:117–21. doi: 10.1080/10245330601111540 17454192

[39] Mielcarek M Leisenring W Torok-Storb B Storb R. Graft-versus-host disease and donor-directed hemagglutinin titers after ABO-mismatched related and unrelated marrow allografts: evidence for a graft-versus-plasma cell effect. Blood. (2000) 96:1150–6. doi: 10.1182/blood.V96.3.1150 10910936

[40] Lee J-H Lee K-H Kim S Lee J-S Kim S-H Kwon S-W. Anti-A isoagglutinin as arisk factor for the development of pure red cell aplasia after major ABO-incompatible allogeneic bone marrow transplantation. Bone Marrow Transpl. (2000) 25:179–84. doi: 10.1038/sj.bmt.1702121 10673677

[41] Schetelig J Breitschaft A Kroger N Zabelina T Ebell W Bornhauser M. After major ABO-mismatched allogeneic hematopoietic progenitor cell transplantation, erythroid engraftment occurs later in patients with donor blood group A than donor blood group B. Transfusion. (2005) 45:779–87. doi: 10.1111/j.1537-2995.2005.04236.x 15847669

[42] Aung FM. Incidence and natural history of pure red cell aplasia in major ABO-mismatched haematopoietic cell transplantation. Br J Haematol. (2013) 160:798–805. doi: 10.1111/bjh.12210 23330820 PMC4078723

[43] Canaani J Savani BN Labopin M Huang X-J Ciceri F Arcese W. Impact of ABO incompatibility on patients' outcome after haploidentical hematopoietic stem cell transplantation for acute myeloid leukemia-a report from the acute leukemia working party of the EBMT. Haematologica. (2017) 102:1066–74. doi: 10.3324/haematol.2016.160804 PMC5451338 28255020

[44] Stussi G Halter J Bucheli E Valli PV Seebach L Gmür L. Prevention of pure red cell aplasia after major or bidirectional ABO blood group incompatible hematopoietic stem cell transplantation by pretransplant reduction of host anti-donor isoagglutinins. Haematologica. (2009) 94:239–48. doi: 10.3324/haematol.13356 PMC2635395 19144657

[45] Lee JH Lee JH Choi SJ Kim S Seol M Kwon SW. Changes of isoagglutinin titres after ABO-incompatible allogeneic stem cell transplantation. Br J Haematol. (2003) 120:702–10. doi: 10.1046/j.1365-2141.2003.04128.x 12588361

[46] Kimura F Kanda J Ishiyama K Yabe T Yoshifuji K Fukuda T. ABO blood type incompatibility lost the unfavorable impact on outcome in unrelated bone marrow transplantation. Bone Marrow Transplant. (2019) 54:1676–85. doi: 10.1038/s41409-019-0496-2 30867557

[47] Tomonari A Takahashi S Ooi J Tsukada N Konuma T Kobayashi T. Impact of ABO incompatibility on engraftment and transfusion requirement after unrelated cord blood transplantation: a single institute experience in Japan. Bone Marrow Transplant. (2007) 40:523–8. doi: 10.1038/sj.bmt.1705765 17646845

[48] Booth GS Savani BN Langston AA. Pure red blood cell aplasia: patient management pitfalls inmajor ABO-incompatible haematopoietic cell transplantation. Br J Haematol. (2021) 193:701–2. doi: 10.1111/bjh.17465 33844846

[49] Voak D Williams MA. An explanation of the failure of the direct antiglobulin test to detect erythrocyte sensitization in ABO haemolytic disease of the newborn and observations on pinocytosis of IgG anti-A antibodies by infant (cord) red cells. Br J Haematol. (1971) 20:9–23. doi: 10.1111/j.1365-2141.1971.tb00782.x 5099693

[50] Lemaire B Combescure C Chalandon Y Vuilleumier N Waldvogel Abramowski S. Kinetics of disappearance and appearance of isoagglutinins A and B after ABO-incompatible hematopoietic stem cell transplantation. Bone Marrow Transplant. (2022) 57:1405–10. doi: 10.1038/s41409-022-01737-z PMC9439946 35752741

[51] Wernet D Mayer G. Isoagglutinins following ABO-incompatible bone marrowtransplantation. Vox Sang. (1992) 62:176–9. doi: 10.1111/j.1423-0410.1992.tb01194.x 1609520

[52] Damodar S George B Mammen J Mathews V Srivastava A Chandy M. Pre-transplant reduction of isohaemagglutinin titres by donor group plasma infusion does not reduce the incidence of pure red cell aplasia in major ABO-mismatched transplants. Bone Marrow Transplant. (2005) 36:233–5. doi: 10.1038/sj.bmt.1705031 15908965

